# Fermentative processes for the upcycling of xylose to xylitol by immobilized cells of *Pichia fermentans* WC1507

**DOI:** 10.3389/fbioe.2024.1339093

**Published:** 2024-01-18

**Authors:** Raffaella Ranieri, Francesco Candeliere, Jaime Moreno-García, Juan Carlos Mauricio, Maddalena Rossi, Stefano Raimondi, Alberto Amaretti

**Affiliations:** ^1^ Department of Life Sciences, University of Modena and Reggio Emilia, Modena, Italy; ^2^ Department of Agricultural Chemistry, Edaphology and Microbiology, University of Cordoba, Cordoba, Spain; ^3^ Biogest-Siteia, University of Modena and Reggio Emilia, Modena, Italy

**Keywords:** xylose, xylitol, immobilized cells, Pichia fermentans, alginate, fungal pellet

## Abstract

Xylitol is a pentose-polyol widely applied in the food and pharmaceutical industry. It can be produced from lignocellulosic biomass, valorizing second-generation feedstocks. Biotechnological production of xylitol requires scalable solutions suitable for industrial scale processes. Immobilized-cells systems offer numerous advantages. Although fungal pellet carriers have gained attention, their application in xylitol production remains unexplored. In this study, the yeast strain *P. fermentans* WC 1507 was employed for xylitol production. The optimal conditions were observed with free-cell cultures at pH above 3.5, low oxygenation, and medium containing (NH_4_)_2_SO_4_ and yeast extract as nitrogen sources (xylitol titer 79.4 g/L, Y_P/S_ 66.3%, and volumetric productivity 1.3 g/L/h). Yeast cells were immobilized using inactive *Aspergillus oryzae* pellet mycelial carrier (MC) and alginate beads (AB) and were tested in flasks over three consecutive production runs. Additionally, the effect of a 0.2% w/v alginate layer, coating the outer surface of the carriers (cMC and cAB, respectively), was examined. While Y_P/S_ values observed with both immobilized and free cells were similar, the immobilized cells exhibited lower final xylitol titer and volumetric productivity, likely due to mass transfer limitations. AB and cAB outperformed MC and cMC. The uncoated AB carriers were tested in a laboratory-scale airlift bioreactor, which demonstrated a progressive increase in xylitol production in a repeated batch process: in the third run, a xylitol titer of 63.0 g/L, Y_P/S_ of 61.5%, and volumetric productivity of 0.52 g/L/h were achieved. This study confirmed *P. fermentans* WC 1507 as a promising strain for xylitol production in both free- and entrapped-cells systems. Considering the performance of the wild strain, a metabolic engineering intervention aiming at further improving the efficiency of xylitol production could be justified. MC and AB proved to be viable supports for cell immobilization, but additional process development is necessary to identify the optimal bioreactor configuration and fermentation conditions.

## 1 Introduction

The transition from a linear fossil-fuel economy to a circular approach necessitates the implementation of industrial strategies that can enhance the utilization of second-generation feedstocks. Within this framework, lignocellulosic biomass (LCB) emerges as a promising resource for closing this loop. LCB residues, approximately 145 billion tons annually, are abundantly available raw materials that do not compete with food production (Haldar et al., 2022). Due to the substantial carbohydrate content, LCB represents a valuable source of fermentable sugars like glucose, arabinose, mannose, and xylose, as well as aromatic compounds. LCB could provide numerous industrially relevant molecules generated by biorefineries, among which sugar alcohols such as xylitol and arabitol have been recognized as top value-added chemicals and have garnered increasing scientific attention (Werpy and Peterson, 2004; Erickson et al., 2012; Raimondi et al., 2022).

Xylitol, a five-carbon sugar alcohol, possesses diabetic-friendly sweetening power, anti-cariogenic properties, and the ability to inhibit microbial growth (Morais Junior et al., 2019). It finds extensive application in food, dental, and pharmaceutical products, with a global market demand of 200 billion tons per year, projected to grow from 2021 to 2026 (Ravella et al., 2022). The bioconversion of xylose into xylitol can be achieved through the metabolic capability of some yeasts: xylitol accumulates when xylose is reduced to xylitol by xylose reductase (XR) but was not further oxidized to xylulose by xylitol dehydrogenase (XDH), in order to be channeled to the to the pentose-phosphate catabolic pathway for energy production (Zha et al., 2021). Notably, the most extensively studied yeasts for this purpose belong to the genera *Debaryomyces* (e.g., *D. nepalensis*, *D. hansenii*), *Candida* (e.g., *C. intermendia*, *C. tropicalis, C. boidinii*, , *C. parapsilosis*), and *Meyerozyma* (*M. gulliermondii* and *M. caribbica*) as well as species like *Barnettozyma populi*, *Scheffersomyces stipitis,* and *Kluyveromyces marxianus* (Oh and Kim, 1998; Kwon et al., 2006; Prakash et al., 2011; Wu et al., 2018; Tamburini et al., 2015; Saha and Kennedy, 2020; Queiroz et al., 2022; Kumar et al., 2022). *Pichia fermentans* deserves to be added to these species (Prabhu et al., 2020), with strain WC 1507 that transforms xylose into xylitol with promising yields and titer (Raimondi et al., 2023).

Embracing biotechnological approaches for value-added product production necessitates the implementation of scalable solutions applicable to industrial-scale processes. In this context, the immobilized-cell systems possess features that make them an interesting solution for scale-up of bioprocesses, such as the ease of biomass recovery and reusability in successive fermentation batches, and the possibility to provide high cells concentration to the processes without affecting the catabolic ability of the biocatalyst (Moreno-Garcia et al., 2018; Chacón-Navarrete et al., 2021). Several immobilization methods have been developed and tested over the years, differing in terms of the localization of cells with respect to the support and the nature of the microenvironment. These include adsorption on a support surface, mechanical containment behind a barrier, self-immobilization, and the physical entrapment in preformed porous support materials or hydrogels (Jain et al., 2023). Among these techniques, the latter is one of the most widely explored. Different materials, such as agar-agar, alginate, alginate-chitosan, κ-carrageenan, polyacrylamide, polyurethane, and polyvinyl-alcohol have been tested as matrices for cell entrapment (Chacón Navarrete et al., 2021).

Out of these, calcium alginate is one of the most explored matrices for cells immobilization because of cost-efficiency, straightforward bead preparation process, and gentle operating conditions (Clements et al., 2014). Nevertheless, alginate-based immobilization systems have the disadvantage of limited mechanical stability and low oxygen diffusion within the polymer matrix, which can restrict the applications (Willaert, 2011). Mycelial carriers, also known as fungal pellets, have increased in attention as a new biomaterial for entrapped-cell fermentations. Filamentous fungi in liquid culture exhibit hyphal growth that can lead to either dispersed mycelium or fungal pellets (Veiter et al., 2018), the latter presenting higher mechanical resilience in bioreactors under gentle agitation. Mycelial carriers have already been successfully used in microalgae entrapment for wastewater treatment (Wrede et al., 2014) and in the production of wine and beer (Moreno-Garcia et al., 2018; Ogawa et al., 2022; Pastor-Vega et al., 2023). To further enhance the performance of immobilized cells on various carriers, several strategies have been explored, including the application of external coatings such as alginate and chitosan. These coatings have demonstrated improvements in terms of mechanical strength, tolerance against inhibitory substances, and reduced cell leakage from the matrix (Willaert, 2011; López-Menchero et al., 2021). Substantial previous research efforts have been spent to develop immobilized cell fermentation processes for xylitol production from lignocellulosic substrates, albeit the application of mycelial carriers as a support for this process remains unexplored and is tested for the first time in this work.

In this study, we focused on improving the conversion of xylose into xylitol by *P. fermentans* WC 1507. Process improvement started investigating the effect of oxygenation, pH, medium composition on the performance of suspended-cells batch cultures. Subsequently, the strain was encapsulated in both alginate and mycelial carriers, each configured in two distinct forms: uncoated and coated with a 0.2% alginate layer. This experimental setup facilitated a comparative analysis of performance over three consecutive runs. Finally, xylitol production using immobilized cells of *P. fermentans* WC 1507 within alginate beads was tested in a laboratory-scale bioreactor configured in an air-lift setup.

## 2 Materials and methods

### 2.1 Strains, media, and culture condition

All the chemicals were purchased from Sigma-Aldrich (Steinheim, Germany) unless otherwise stated.

The yeast strain *P. fermentans* WC 1507, belonging the collection of the Laboratory of Microbial Biotechnologies (Department of Life Sciences, University of Modena and Reggio Emilia) and already known for its xylose-to-xylitol conversion ability (Raimondi et al., 2023), was routinely cultured in YPD (20 g/L glucose, 10 g/L tryptone, 10 g/L yeast extract) broth at 30°C in aerobic conditions. The inoculum for free cells fermentation experiments and the biomass for immobilized-cells processes were prepared in MY medium (20 g/L xylose, 3 g/L yeast extract, 2 g/L (NH_4_)_2_SO_4_, 3 g/L KH_2_PO_4_, 1 g/L K_2_HPO_4_, and 1 g/L MgSO_4_ × 7H_2_O), where the yeast was incubated aerobically at 30°C. Unless otherwise specified, xylitol production was conducted in a xylose-enriched MY medium containing 120 g/L of xylose. Any modifications required for specific experiments were explicitly mentioned.

*Aspergillus oryzae* FST 76–2, which is part of the collection of the Department of Agricultural Chemistry, Edaphology, and Microbiology at the University of Cordoba (Spain), was used to produce mycelium pellets for yeast immobilization. The strain was routinely cultured at 28°C on agar plates containing SM medium (17 g/L corn meal agar, 1 g/L yeast extract, 2 g/L glucose, 20 g/L agar). Mycelial pellets were generated using fungal pellet medium (FPM) consisting of 60 g/L glucose, 3 g/L yeast extract, 3 g/L NaNO_3_, 1 g/L K_2_HPO_4_, 10.2 g/L MgSO_4_ ∙ 7 H_2_O, 0.5 g/L KCl, 0.02 g/L FeSO_4_ ∙ 7 H_2_O, pH = 5.5.

### 2.2 Xylitol production by free cells of *P. fermentans* WC 1507

Bioreactor batch fermentations with suspended cells of *P. fermentans* WC 1507 were carried out to determine the effect of medium composition (i.e., xylose and ammonium concentration) and process parameters (i.e., the pH and oxygenation) on xylose-to-xylitol transformation. Fermentation runs were performed in 500 mL stirrer tank bioreactors (Mini Bio, Applikon Biotechnology, Delft, the Netherlands), filled with 350 mL of xylose enriched MY medium and inoculated (5%, v/v) with an overnight grown seed culture. Cultures were maintained at 32.5°C and aerated with 0.5 v/v/min filter-sterilized air. Foaming was prevented by the addition of 150 μL of a mixture (1:1, v/v) of Xiameter 1,520 (Dow Corning, Midland, MI, United States of America) and polypropylene-glycol.

In the MY medium, xylose was utilized at the concentration of 90, 120, and 150 g/L, with or without (NH_4_)_2_SO_4_. To investigate the effect of the oxygenation on the xylose-to-xylitol conversion, two different conditions were compared: i) dissolved oxygen tension (DOT) higher than 20%, obtained by cascade-controlled stirring between 1,100 and 1700 rpm; ii) free DOT, obtained applying constant stirring at 1,100 rpm. To study the effect of pH on the bioconversion performance, four different pH conditions were tested: pH higher than 2.5, 3.5, 4.5, and 5.5, with pH decrease prevented by the automatic addition of 1 M NaOH. The stirring was constant at 1,100 rpm and the process was monitored for 144 h. All the processes were periodically sampled to determine growth, xylose consumption, and xylitol generation.

### 2.3 Yeast immobilization

The biomass of *P. fermentans* WC 1507, cultured for 24 h in MY medium with 20 g/L xylose, was entrapped in alginate beads (AB) and in mycelial carrier (MC), and alginate-coated AB and MC (hereinafter referred to as cAB and cMC).

To produce the AB, the culture of *P. fermentans* WC 1507 was mixed (1:1 v/v) with 40 g/L sodium alginate. The suspension was pumped with a peristaltic pump and dropped through a syringe needle into a gently stirred 20 g/L CaCl_2_ solution. The AB were kept in the calcium solution at room temperature for 30 min, then they were washed twice with water and stored in physiological solution (9 g/L NaCl) at 4°C. According to the initial concentration, alginate 2% (w/v) AB were obtained.

The support for immobilization in MC consisted of inactivated mycelial pellets of *A. oryzae* FST 76–2. To produce the pellets, the fungal spores from an 8-days grown SM plate were suspended in 1 mL of FPM, vortexed, sonicated in a water bath for 10 min, and inoculated at the concentration of 6 ×10^3^ spores/mL in a 1 L flasks containing 250 mL of FPM. After 3 days of aerobic incubation at 28°C, the mycelial granules or pellets were washed with water, and sterilized in autoclave for inactivation. The mycelial pellets, the cells of *P. fermentans* WC 1507 previously collected by centrifugation, and sterile water were mixed with the ratio of 1:1:5 (w/w/v) and vortexed. To force the yeast cells towards the core of the fungal pellets, the suspension was subjected to vacuum for 10 s, then the MC were washed with water and stored in physiological solution at 4°C (Lúquez-Caravaca et al., 2023).

To produce cAB and cMC, 30 g of AB or MC were submerged in 2 g/L sodium alginate for 5 min, drained in a sterile sieve, and rinsed with 200 mL of sterile water. Hydrogel film coating was obtained transferring the beads into a gently stirred 20 g/L CaCl_2_ solution. After 30 min at room temperature, the cAB and cMC were washed with water and stored in physiological solution at 4°C. Photographs of the carrier beads were acquired and analyzed utilizing ImajeJ (https://imagej.net/), in order to measure their size and monitoring swelling during the bioconversion.

### 2.4 Xylitol production by immobilized cells of *P. fermentans* WC 1507

Xylitol production by immobilized cells of *P. fermentans* WC 1507 was evaluated in flasks experiments. 2.5 g of immobilized yeast cells were added to 100 mL flasks filled with 25 mL of MY medium containing 120 g/L xylose. The flasks were incubated at 200 rpm in an orbital shaker at 28°C for 116 h. Aliquots of medium were collected every 24 h to analyze xylose and to monitor pH and cells release.

To determine the reusability across consecutive production runs, the immobilized biomass was recovered and pooled at the end of the incubation, washed with sterile water, and added to fresh medium to begin anew. At the end of each run, some immobilized biomass was withdrawn to determine the number of entrapped cells (cells/g carrier), cells vitality (%), and beads swelling (mm). Five replicate flasks were set up for the first run and thus decreased to three replicates for the third one.

Bioreactor batch fermentations with immobilized cells of *P. fermentans* WC 1507 were carried out in a laboratory-scale airlift bioreactor. The apparatus was set up by modifying the Mini Bio apparatus, with the removal of the stirring shaft and the installation of a draft tube with a diameter of 30 mm. The process was performed in 350 mL of MY medium with 120 g/L of xylose and 35 g of AB loaded with approx. 2.0 ✕10^8^ yeast cells per g. Mixing was realized by providing the culture with 4 v/v/m of air. The pH was kept at values >3.5 by automatic titration with 1 m NaOH. Aliquots of medium were collected every 24 h to analyze xylose and to monitor cells releasing.

### 2.5 Biological and chemical analysis

To quantify xylose and xylitol concentration, samples were clarified by centrifugation (9,500 xg for 5 min) and analyzed by HPLC (1,200 System, Agilent Technologies, Waldbronn, Germany) equipped with a refractive index detector. Isocratic elution was carried out at 60°C with 0.6 mL/min of 5 mM H_2_SO_4_ through an ion exclusion column (Aminex HPX-87 H, Bio-Rad, Hercules, CA, United States of America), according to Amaretti et al. (2018). The xylose to xylitol conversion yield (Y_P/S_) was calculated on mass basis as the ratio between xylitol produced and xylose consumed (Amaretti et al., 2020). Volumetric productivity was calculated by dividing xylitol titer by time of fermentation (hours).

To quantify the free cells in the supernatant or those entrapped in the carriers, after dissolution or disruption, the yeast cells were counted in Thoma’s chamber and assayed for vitality. For alginate beads, 1 g of AB or cAB was placed in 9 mL of 10 g/L sodium-citrate solution and vortexed until complete dissolution. For disruption of mycelium carrier, 1 g of MC or cMC was broken with a tissue grinder in 9 mL of physiological solution or 10 g/L sodium-citrate solution, respectively. Vitality was evaluated by mixing (1:1, v/v) the cells suspension and a solution containing 0.1 g/L methylene blue and 20 g/L dihydrate sodium citrate. After 5 min incubation at room temperature, at least 200 cells were observed by microscope, the colorless and the blue ones being considered vital and non-vital, respectively (Kwolek-Mirek and Zadrag-Tecza, 2014).

### 2.6 Statistical analysis

All the reported values are means of at least three separate experiments. t-test and ANOVA followed by Tukey *post hoc* analysis were utilized for the comparison of means. Differences were considered statistically significant for *p* < 0.05.

## 3 Results and discussion

### 3.1 Xylitol production by suspended cultures of *P. fermentans* WC 1507

**FIGURE 1:**
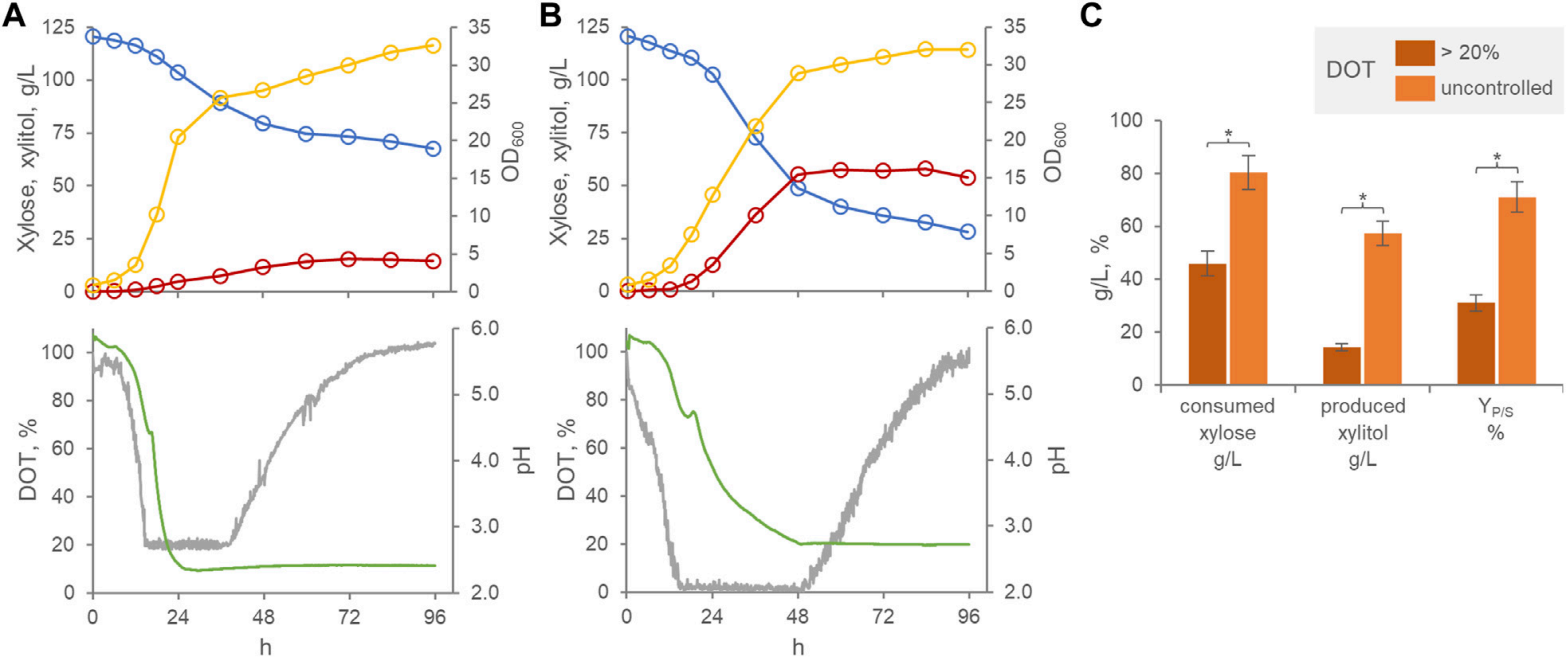
Effect of oxygenation on the transformation of xylose to xylitol by *P. fermentans* WC 1507. Time course of batch cultures with DOT kept >20% **(A)** and with uncontrolled DOT **(B)**. Representative runs of fermentation processes carried out in triplicate. Colors: xylose, cyan; Xylitol, red; OD600, yellow; pH, green; DOT, grey. **(C)** Mean values of xylose consumed, xylitol produced and conversion yield at 60 h are reported. Values are means (n = 3, SD always <10%), * indicating a significant difference (*p* < 0.05, t-test).

When cultured batch-wise in a xylose-based medium within a stirred tank bioreactor, *P. fermentans* WC 1507 produced xylitol essentially during the growth phase, confirming a previous study assessing the ability of the strain in preliminary shake-flasks experiments (Raimondi et al., 2023) (Figure 1A,B). The application of cascade control of stirring, aimed at preventing the occurrence of oxygen transport limitations, resulted in an improved growth rate without affecting the final turbidity of the culture. On the other hand, improved oxygenation depressed both xylose consumption and xylitol production. Under limited oxygenation conditions, at the entrance into the stationary phase after 48 h, 80.5 g/L xylose were consumed and yielded 57.3 g/L xylitol, with a Y_P/S_ of 71%. When the DOT was kept >20%, significantly lower values (*p* < 0.05) were achieved, 45.9 g/L xylose being consumed to yield 14.3 g/L xylitol, with a Y_P/S_ of 31% (Figure 1C). This result confirmed previous evidence indicating microaerophilic conditions as the most appropriate for xylitol production by other yeast species. Yeasts can use xylose for cellular respiration in aerobic conditions. After entering the cell, a xylose reductase NAD(P)H-dependent reduces it to xylitol. While the excess of produced xylitol can be excreted out of the cells, the remain is oxidized to 5-xylulose through a xylitol dehydrogenase NADP^+^-dependent, then 5-xylulose can be metabolized through the pentose phosphate pathway and glycolysis (Moysés et al., 2016; Zha et al., 2021). When an excess of oxygen occurs, NADP^+^/NAPDH ratio increases and, therefore, intensify the xylitol-to-xylulose conversion, reducing the accumulation and excretion of xylitol.

**FIGURE 2:**
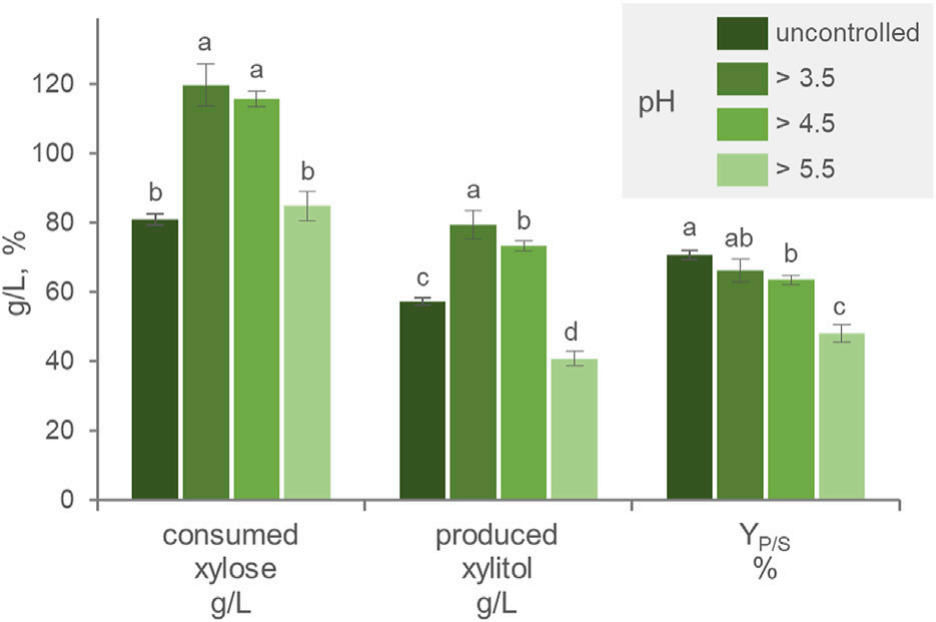
Effect of the pH on the transformation of xylose to xylitol by batch cultures of *P. fermentans* WC 1507. The pH was uncontrolled or kept above 3.5, 4.5, and 5.5. The values of xylose consumed, xylitol produced and Y_P/S_ after 60 h of fermentation are reported. Values are means ± SD (n = 3). Within each series, means with different letters significantly differ (*p* < 0.05, ANOVA, Tukey *post hoc*).

Throughout the growth and production phase, the pH of the culture progressively decreased to 2.7. The effect of pH was thus investigated, by automatically titrating acidity and preventing the pH to drop below 3.4, 4.5, or 5.5. Compared with the free-pH culture, pH values >3.5 and 4.5 maximized xylose consumption, leading the substrate to get nearly exhausted during cultivation (Figure 2). Xylitol generation was the highest (*p* < 0.05) in cultures with the pH > 3.5, that reached 79.4 g/L xylitol, a value that was slightly but significantly higher than that obtained by cultures with the pH > 4.5 (73.4 g/L). The cultures with pH > 5.5 were the less efficient in producing xylitol, yielding the lowest titer (40.8 g/L). The cultures with uncontrolled pH presented the highest Y_P/S_, followed in decreasing order by those with pH > 3.5, 4.5, and 5.5. These results suggested that acidic pH was necessary for xylitol production and that progressively higher values had the effect of decreasing the efficiency of the transformation. Yeasts have been observed to produce xylitol across a broad pH range, with high pH that negatively influences the activity of the xylose membrane transporter, while an acid environment can affect the cell maintenance requirement as well as the redox balance of the bioreduction (Converti and Dominiguez, 2001; Dasgupta et al., 2017). In the context of second-generation biorefinery development, xylitol production can be attained through the utilization of lignocellulosic hydrolysates as a feedstock (Vanmarcke et al., 2021). These substrates may be characterized by the presence of inhibitory molecules, such as acetic acid, and it is expected that the acidity of the environment could lead to increased acetate toxicity (Trček et al., 2015), potentially resulting in a shift in pH preference towards a less acidic range. To achieve an optimal xylitol titer, an overall balance among these factors is necessary and should be investigated, preferably also using real industrial feedstock.

**FIGURE 3:**
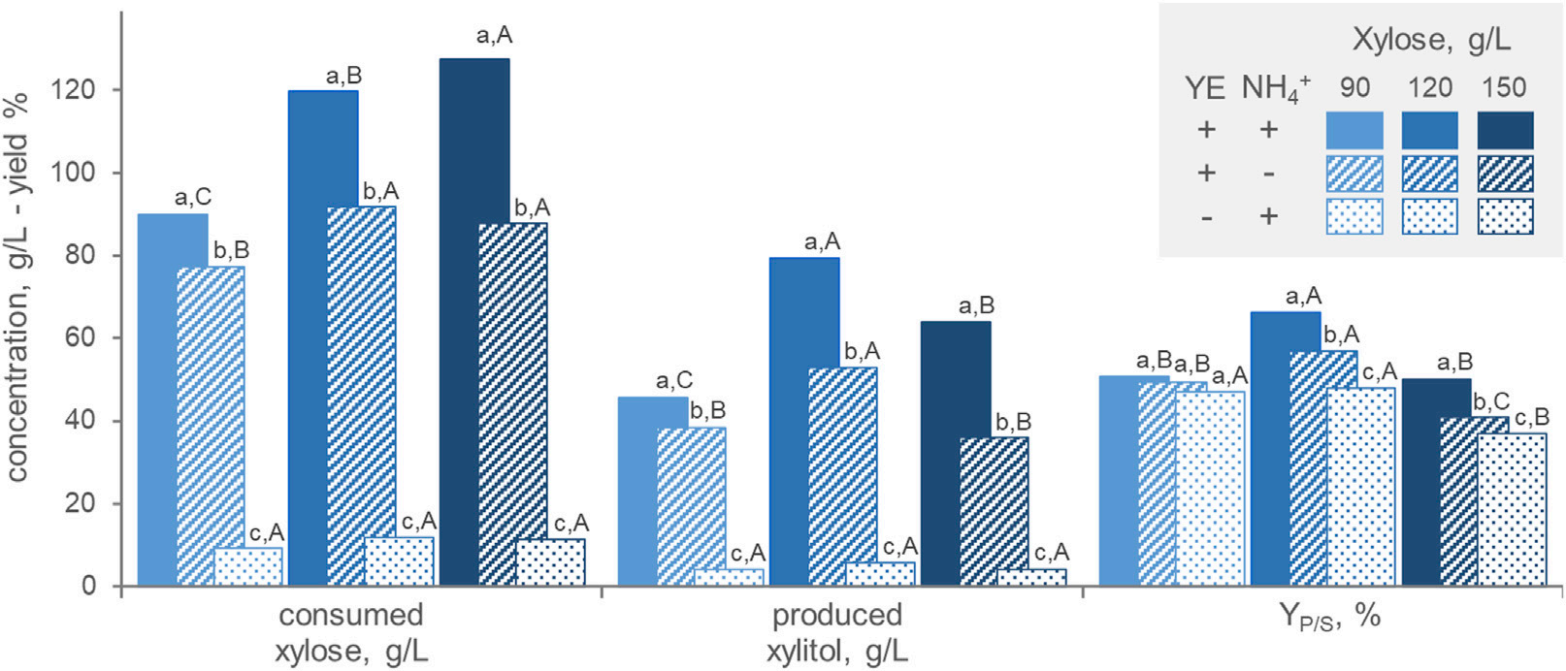
Effect of the initial xylose concentration and the presence of inorganic (NH_4_^+^) and organic (yeast extract, YE) nitrogen on the transformation of xylose to xylitol by *P. fermentans* WC 1507. The values of xylose consumed, xylitol produced and conversion yield after 60 h (for 90 and 120 g/L xylose) or 80 h (for 150 g/L xylose) are reported. Values are means (n = 3, SD always <10%); Within each series, means with different letters significantly differ (*p* < 0.05, ANOVA, Tukey *post hoc*), the lowercase and uppercase ones being utilized for comparisons among nitrogen sources and xylose concentrations, respectively.

In order to assess the medium composition, batch fermentations were carried out in the presence/absence of ammonium and yeast extract as nitrogen source and with different initial xylose concentrations (90, 120, and 150 g/L) (Figure 3). At all initial xylose concentrations, the highest amounts of both consumed xylose and generated xylitol were obtained with the organic nitrogen source (*p* < 0.05), that had a cumulative effect with the inorganic one. These results confirm that xylitol production by *P. fermentans* WC 1507 goes along with growth and that nitrogen restriction is detrimental for production. On the other hand, high xylose concentration is necessary for xylitol generation, that reached its maximum, in terms of substrate consumption, product generation, and Y_P/S_ when the culture was grown with 120 g/L. Growth under microaerophilic conditions may force the strain to reduce a great amount of xylose to xylitol, in order to oxidize part of the reduced cofactors generated with the catabolism. However, *P. fermentans* WC 1507 did not grow nor produce xylitol in anaerobiosis (data not shown), indicating that the strain relies on respiration to gain energy for its cellular processes.

Among the various conditions described in this study, the most successful process resulted in a xylitol titer of 79.4 g/L, with a Y_P/S_ of 66.3%, and a volumetric productivity of 1.3 g/L/h. Except for the processes exploiting *C. tropicalis*, which, to the best of our knowledge, have shown the best performance thus far (Oh and Kim, 1998; Kwon et al., 2006), the batch process described in this study achieved among the highest values of xylitol titer, Y_P/S_, and volumetric productivity compared to other batch processes involving non-engineered yeast strains (Table 1). In particular, *P. fermentans* WC 1507 outperformed the wild-type *P. fermentans* used in a similar process by Prabhu et al. (2020) and exhibited performance similar to the selected mutant obtained through random chemical mutagenesis by the same authors. Therefore, the strain *P. fermentans* WC 1507 could represent a promising starting point for both strain and process improvement aiming at further enhancing the ability of this yeast.

**TABLE 1:** Fermentative performance of non-engineered yeasts transforming pure xylose into xylitol. Product yield pertaining to this study was calculated considering the initial amount of substrate.

Strain		Xylitol titre (g/L)	Y_P/S_ (%)	Volumetricproductivity (g/L/h)	Time (h)	Initial xylose (g/L)	References
*B. populi* NRRL Y-12728		58	71	0.43	136	100	Saha and Kennedy (2020)
*C. intermedia* FL023		46	57	0.38	120	80	Wu et al. (2018)
*C. tropicalis* KCTC 10457		185	87	3.66	47	200	Kwon et al. (2006)
*C. tropicalis* KFCC 10960		225	90	1.44	156	250	Oh and Kim (1998)
*D. hansenii*		69	76	0.44	156	100	Prakash et al. (2011)
*D. hansenii* NRRLY-7426		107	84	1.48	72	130	Dominguez (1998)
*D. nepalensis* NCYC 3413		79	-	0.82	96	100	Kumar et al. (2022)
*M. caribbica* 5XY2		20	60	0.16	120	50	Sukpipat et al. (2017)
*P. fermentans*	w.t.	17	57	0.35	48	30	Prabhu et al. (2020)
	mutant	99	67	0.59	168	150	
*P. fermentans* WC1507		79	66	1.3	60	120	This study

### 3.2 Xylitol production by immobilized cells in mycelial and alginate carriers

**FIGURE 4:**
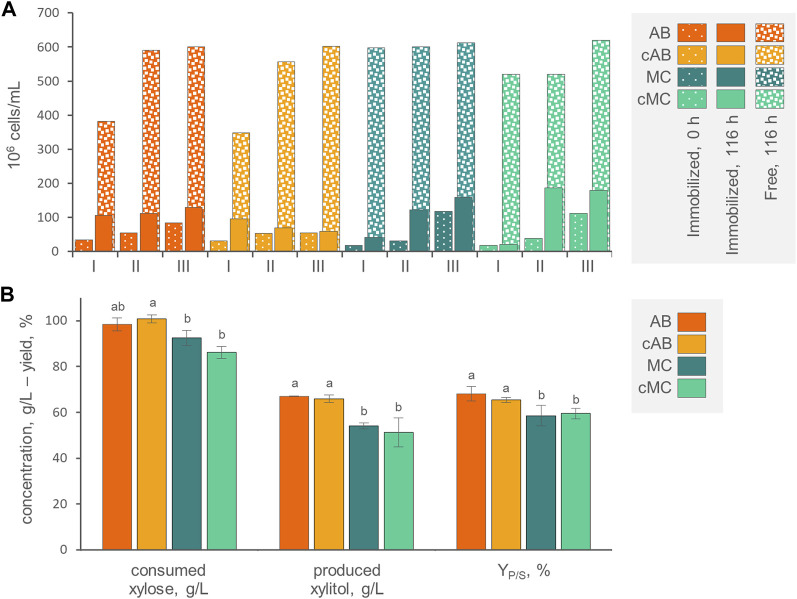
**(A)** Counts of entrapped and free cells at the beginning and at the end of three consecutive runs with AB, cAB, MC, and cMC. Values are means (n = 3, SD always <10%). The percentage of entrapped cells with respect to total cells is reported. **(B)** Values of xylose consumed, xylitol produced and conversion yield, obtained by AB, cAB, MC, and cMC at the end of the third run, after 116 h of incubation. Values are means ± SD (n = 3). Within each series, means with different letters significantly differ (*p* < 0.05, ANOVA, Tukey *post hoc*).

Since xylitol production by *P. fermentans* WC 1507 presented limited oxygen demand, it was well suited to be carried out with immobilized cells, also offering the advantage of possible reuse of the biomass. Immobilized cells of the yeast, in the forms AB, MC, cAB, and cMC, were employed for three consecutive runs in flasks of MY medium containing 120 g/L xylose (Figure 4). The first run was started with 1.3–2.1 × 10^7^ entrapped cells per g, resulting in asuspension of 1.8–3.4 × 10^7^ immobilized yeasts per mL of culture. The swelling of the beads and the count of free and entrapped cells were evaluated at the beginning and at the end of each run (Figure 4A; Supplementary Figure S1).

At the end of each incubation run, the counts of cells entrapped within AB, MC, cAB, and cMC were significantly more numerous (*p* < 0.05) compared to the initial counts, determining a progressive increase of entrapped cells. For instance, the 3^rd^ run initiated with 5.4–11.7 × 10^7^ immobilized yeasts per mL of culture (Figure 4A). This effect was more evident for mycelium immobilized systems MC and cMC, where immobilized cells became significantly more numerous than in alginate beads, reaching up to 1.8 × 10^8^ cells/mL. At each run, the increase of entrapped cells was always accompanied by the release of yeasts in the medium. The cells directly liberated by the carriers or subsequently grown in the medium reached the concentration of 6.0–6.2 × 10^8^ cells/mL at the end of the third run. At this time point, the cells entrapped in AB, MC, cAB, and cMC represented 17.7%, 9.0%, 20.1%, and 22.5% of total cells. The alginate beads exhibited progressive swelling over the course of the three runs, starting with a mean diameter of 3.4 mm and reaching 5.1 mm. The coating of AB and MC exerted a slight but significant effect on preventing the release of yeasts only in the first two runs. This effect tended to attenuate, and no significant differences were observed at the end of the third run in both alginate and mycelium-based systems.

The kinetics of xylose consumption and xylitol generation by immobilized cells were linear (Supplementary Figure S2). In agreement with previous observations (Dominguez, 1998), with all the carriers and particularly with AB and cAB, the first run was the less efficient in terms of conversion rate, xylose consumption, and xylitol generation, while the second and the third ones proceeded at higher similar rates. At the end of the third run, the AB and cAB performed better (*p* < 0.05) than MC and cMC in terms of xylose consumption, xylitol generation and Y_P/S_ (Figure 4B). After 116 h of incubation, 102.5 g/L xylose was consumed by *P. fermentans* WC 1507 immobilized in AB and cAB, yielding 63.0 g/L xylitol with a Y_P/S_ of 61.5%.

While the Y_P/S_ values observed with both immobilized and free cells were similar, the immobilized cells exhibited lower final xylitol titer and volumetric productivity. Presumably, mass transfer limitations impaired the diffusion of the substrate in the beads. Literature reports cases where xylitol production was more efficient with the cells immobilized within calcium alginate compared with the free ones (Dominguez, 1998; Prakash et al., 2011; Soleimani and Tabil, 2013). An accurate comparison between free and immobilized cells and between our study and previous ones is challenging due to variations in experimental setups. Therefore, operational parameters such as the loading of cells, the size of beads, and the number of reutilization cycles should be specific targets for process improvement. The addition of an outer layer coating the carriers, specifically in cAB and cMC, neither improved nor reduced the bioconversion performances. The film likely did not introduce additional limitations to substrate mass transfer, opening up the perspective to exploit this strategy to enhance the tolerance of immobilized cells against inhibitory substances (Willaert, 2011; López-Menchero et al., 2021).

### 3.3 Application of AB with *P. fermentans* WC 1507 at the bioreactor-scale

**FIGURE 5:**
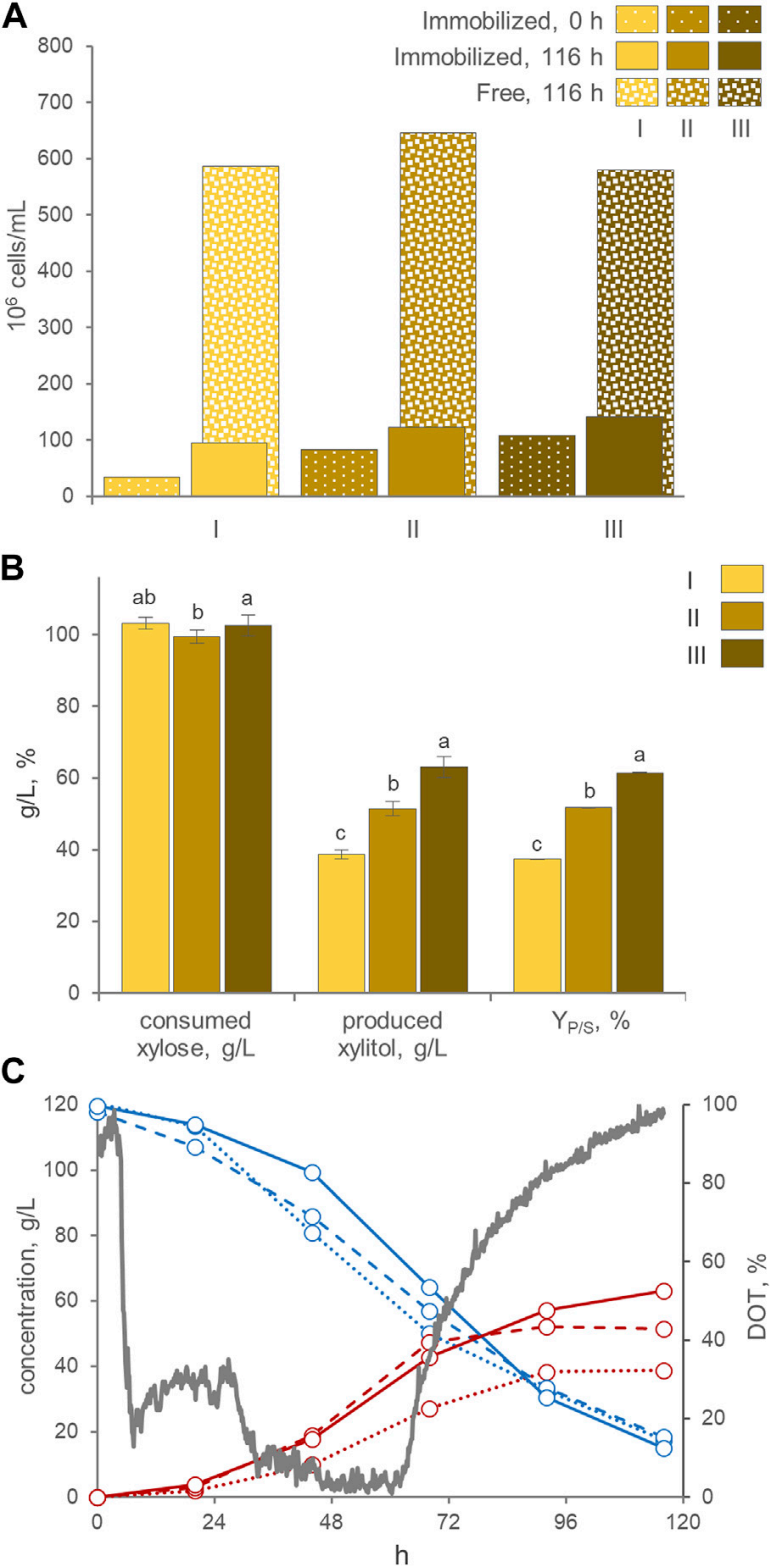
**(A)** Counts of entrapped and free cells at the beginning and the end of three consecutive runs with AB in the air-lift bioreactor (means; n = 3; SD always <10%). The percentage of entrapped cells with respect to total cells is reported. **(B)** Values of xylose consumed, xylitol produced, and conversion yield, obtained at the end of each run, after 116 h of incubation. Values are means ± SD (n = 3). Within each series, means with different letters significantly differ (*p* < 0.05, ANOVA, Tukey *post hoc*). **(C)** Time course of xylose (cyan), xylitol (red), and DOT (grey) in fermentation experiments with cells of *P. fermentans* WC 1507, entrapped in AB. Immobilized cells were utilized in three consecutive runs (I, II, and III, displayed with dotted, dashed, and solid lines, respectively); only the DOT of run III is reported.

AB immobilization was selected for utilization in laboratory-scale bioreactor experiments. The stirred tank bioreactor resulted inadequate to preserve the integrity of the beads (data not shown), therefore the bioreactor was modified into an airlift configuration. The best process parameters (*i.e.*, the pH > 3.5 and the absence of oxygen control) and medium composition (120 g/L xylose, 3 g/L yeast extract, and 2 g/L ammonium sulfate) previously identified in batch cultures were utilized. Three consecutive runs were carried out with the same AB beads. Between each run, the exhausted medium was replaced with new fresh medium. At the start of the first run, 3.4 × 10^7^ immobilized cells/mL were loaded. Throughout the experimental runs, a gradual rise in entrapped cells was observed, concomitant with an increase in the number of free cells (Figure 5A). This trend resulted in entrapped cells comprising 12%–17% of the total cells in the culture by the end of each run. These distribution among entrapped and free cells were similar to those observed in shake flasks. The alginate beads displayed a gradual increase in size during all three runs, exhibiting a progressive swelling phenomenon. The average AB diameter after these runs reached 5.2 mm, similar (*p* > 0.05) to the size achieved in the shake flasks experiment.

Likewise flasks experiments, the three consecutive runs yielded a progressive increase in xylitol production, that passed from a final titer of 38.7 g/L, generated in the first run with a Y_P/S_ of 37.5% and volumetric productivity of 0.33 g/L/h, to a titer of 63.0 g/L xylitol, generated in the third run with a Y_P/S_ of 61.5% and a volumetric productivity of 0.52 g/L/h (Figure 5B). Nonetheless, unlike in flasks experiments, the conversion in the laboratory scale airlift reactor did not proceed linearly, but presented a sigmoidal trend, with xylitol production progressively decelerating and approaching a plateau between 96 and 116 h of incubation, even though around 30–38 g/L of xylose were still available (Figure 5C). The bioreactor likely performed less effectively than flasks because of oxygenation, since the DOT reached values below 10% only at 32 h and recommenced to increase toward saturation at approx. 60 h. When a lower air flow was applied to achieve a DOT close to 0%, as required for xylitol production, it proved inadequate to maintain the immobilized biomass in suspension and circulating within the airlift system. Therefore, further experiments are necessary to find the optimal aeration condition and the airlift design, in order to guarantee an adequate culture mixing and, at the same time, the suboptimal oxygenation levels necessary for xylitol production.

## 4 Conclusion

The strain *P. fermentans* WC 1507 was confirmed as a promising xylitol producer from xylose. Under the optimal process parameters and medium composition, an efficient batch process with free cells was developed, achieving among the highest xylose titers, conversion yields, and productivities ever reported with non-recombinant yeast strains. The strain deserves metabolic engineering interventions aimed at further improving the efficiency of xylitol production. The best-performing batch process represents a starting point for further process development, that may include the application of fed-batch approaches or solutions employing reusable highly concentrated cells. With this latter aim, the preliminary processes with immobilized *P. fermentans* WC 1507 cells herein described indicated lower performance compared to free cells. Anyhow, the obtained yields suggest rooms for improvement, and in the context of advancing second-generation biorefineries, a comprehensive study is required to investigate the behavior of immobilized cells using an industrial source of xylose. The focus should be on the enhancement of tolerance against inhibitory substances, primarily arising from the hydrolysis of lignocellulosic biomass.

## Data Availability

The original contributions presented in the study are included in the article/Supplementary Material, further inquiries can be directed to the corresponding author.
