# Supplementary material for: Fermentative processes for the upcycling of xylose to xylitol by immobilized cells of Pichia fermentans WC1507
Source: Front Bioeng Biotechnol. 2024 Jan 18;12:1339093. doi: 10.3389/fbioe.2024.1339093 (PMC10830724; doi:10.3389/fbioe.2024.1339093)
Supplement: Supplementary file 1 [file DataSheet1.docx]

Fermentative processes for the upcycling of xylose to xylitol by immobilized cells of *Pichia fermentans* WC1507

Raffaella Ranieri^1^, Francesco Candeliere^1^, Jaime Moreno-Garcia^2^, Juan Carlos Mauricio^2^, Maddalena Rossi^1,3^, Stefano Raimondi^1,3^, Alberto Amaretti^1,3^

^1^Department of Life Sciences, University of Modena and Reggio Emilia, 41125 Modena, Italy

^2^Deparment of Agricultural Sciences, Edaphology and Microbiology, University of Cordoba, Spain

^3^Biogest-Siteia, University of Modena and Reggio Emilia, Reggio Emilia, Italy

*** Correspondence:**Corresponding Author
stefano.raimondi@unimore.it

Supplementary material

**Supplementary Figure S1.** Diameter (A) and counts of entrapped cells (B) within freshly prepared carriers (0) and the end of three consecutive runs (I, II, and III). Values are means ± SD, n = 3; within each series, means with different letter significantly differ (P < 0.05, ANOVA, Tukey *post hoc*)

**Supplementary Figure S2.** Time course of xylose (cyan) and xylitol (red) concentration in fermentation experiments with cells of *P. fermentans* WC 1507, entrapped in AB, cAB, MC, and cMC. Immobilized cells were utilized in three consecutive runs (I, II, III, displayed with dotted, dashed, and solid lines, respectively). Values are means, n = 3, SD always < 10%.
